# Cross-Reactivity of IgG Antibodies and Virus Neutralization in mRNA-Vaccinated People Against Wild-Type SARS-CoV-2 and the Five Most Common SARS-CoV-2 Variants of Concern

**DOI:** 10.3389/fimmu.2022.915034

**Published:** 2022-06-15

**Authors:** Mandy Schwarze, Andor Krizsan, Alexandra Brakel, Fabian Pohl, Daniela Volke, Ralf Hoffmann

**Affiliations:** ^1^Institute of Bioanalytical Chemistry, Faculty of Chemistry and Mineralogy, Universität Leipzig, Leipzig, Germany; ^2^Center for Biotechnology and Biomedicine, Universität Leipzig, Leipzig, Germany

**Keywords:** angiotensin-converting enzyme-2 (ACE-2), BNT162b2, ELISA, mRNA-1273, receptor-binding domain (RBD), SARS-CoV-2

## Abstract

The rapid development, approval, and production of vaccines against the severe acute respiratory syndrome coronavirus 2 (SARS-CoV-2) in less than 1 year after the first reports of a new infectious disease was a real game changer, providing 80%–90% efficacy in preventing severe etiopathologies of the coronavirus disease 2019 (COVID-19). These vaccines induce an immune response against the SARS-CoV-2 spike (S) protein located on the surface of the virus particle. Antibodies (Abs) recognizing the S-protein can inhibit binding of the virus *via* the S-protein to the angiotensin-converting enzyme-2 (ACE-2) receptor expressed on different human cells, especially when these Abs bind to the interaction site, the so-called receptor-binding domain (RBD). We have expressed the RBDs of wild-type SARS-CoV-2 and five variants of concern (VOCs) to test the immune response in people before vaccination with mRNA vaccines BNT162b2 and mRNA-1273 and after up to three vaccinations using in-house ELISA and inhibition assays. The methods of both assays are provided. Both vaccines initiated similarly high IgG titers after two vaccinations against the wild-type and even two VOC-RBDs (alpha and delta) and strongly inhibited the corresponding RBD-ACE-2 binding. The IgG titers and inhibition of ACE-2 binding were lower for beta and gamma RBDs and much lower for omicron RBD. The third vaccination after 6 months strongly increased both the IgG titers and the neutralizing effect against all variants, especially for omicron, leading to 63% ± 13% neutralization potential. Importantly, neutralization linearly increased with the IgG titers.

## Introduction

Identification of the severe acute respiratory syndrome coronavirus 2 (SARS-CoV-2) in Wuhan in late 2019 and the rapid global spreading due to delayed and ineffective measures triggered an unprecedented pandemic situation within weeks that has persisted for more than 2 years so far (1). Genome sequences of this original SARS-CoV-2 variant, reported shortly after identification of the virus, were immediately used for the design and consecutive development of vaccines, which relied on the wild-type (wt) sequence of the SARS-CoV-2 spike (S) protein. These vaccines were clinically approved at an extraordinary speed (2), allowing the first vaccination programs to start in late 2020, i.e., less than a year after identifying the disease.

The S-protein, which is the largest of the four major structural proteins found in coronaviruses, assembles into a homotrimer with each monomer consisting of a ~670-residue-long N-terminal S1 region located on the outer surface of the virus particle and a ~590-residue-long C-terminal S2 region. Considering the SARS-CoV-2 infection cycle in humans, the S-protein located at the outer surface of the virus binds *via* the S1 region to the angiotensin-converting enzyme-2 (ACE-2) receptor of a host cell, initiating the fusion of viral and host membranes *via* the S2 region, allowing the virus to enter the cell and finally to replicate (3, 4). The receptor-binding domain (RBD) comprises 222 residues, around one-third of the S1 region (2, 3), which binds to the ACE-2 receptor on the surface of the host cell in the first step of viral entry, initiating the infection cycle. Antibodies (Abs) binding to this domain most likely have a pronounced neutralizing effect (3, 5), although epitopes of the S1-protein outside the RBD have also been reported to be neutralizing (5, 6).

The rapid spread of the virus and especially the high vaccination rates obtained in many countries lead to a broad immunity in the population. Expectedly, this evolutionary pressure on the wt SARS-CoV-2 triggered mutations in immunodominant epitopes to escape the immune system in recovered and vaccinated people (7), as shown for Ab therapies (8, 9). Consequently, SARS-CoV-2 variants with escape mutations emerged with a few having the additional evolutionary advantage of higher infection rates, such as alpha (B.1.1.7), beta (B.1.351), gamma (B.1.1.28), delta (B.1.617.1), and most recently the omicron (B.1.1.529) variants (1, 10, 11).

**Figure 1:**
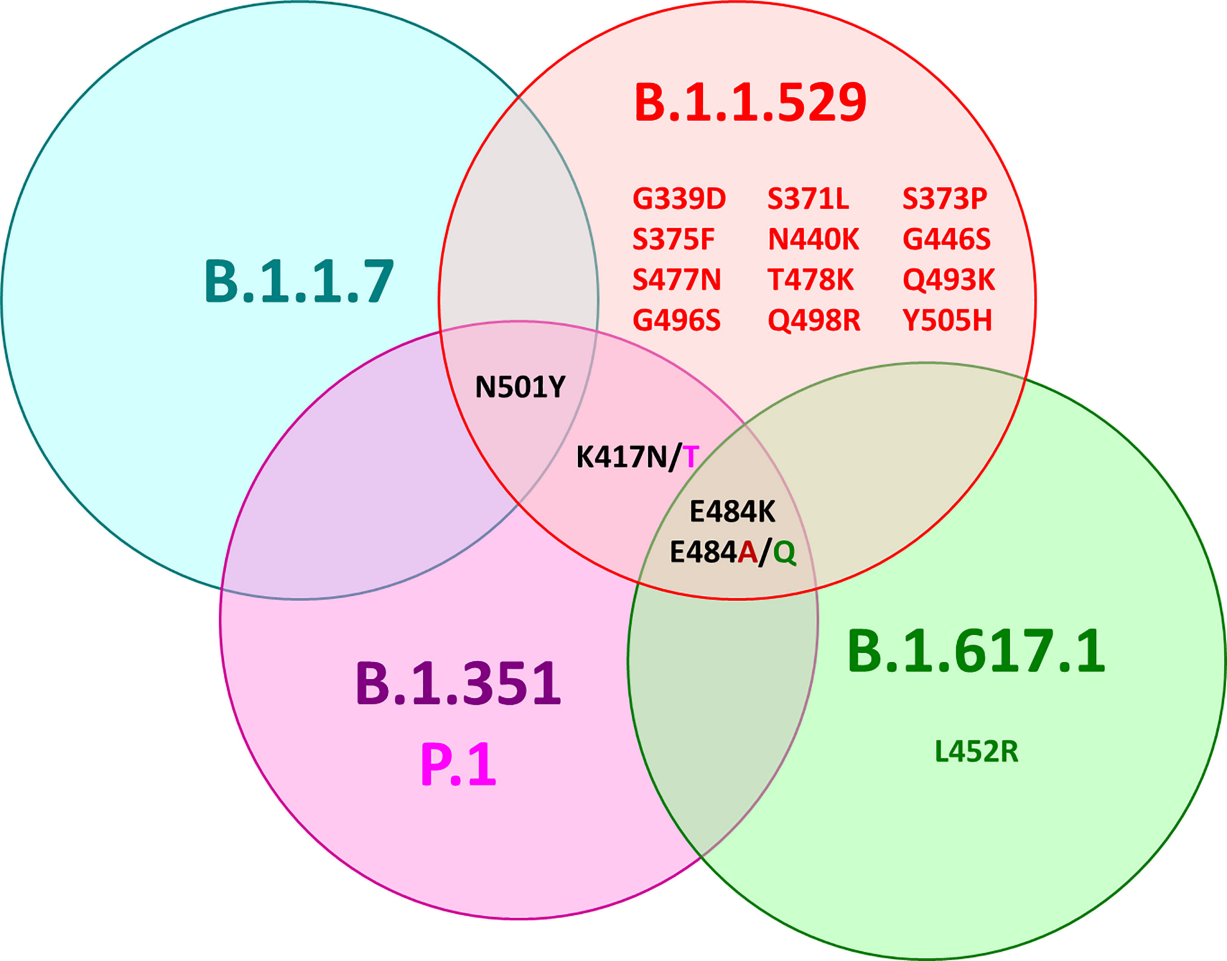
Venn diagram of the mutation sites in the RBDs of alpha (B.1.1.7, blue), beta (B.1.351, purple), gamma (B.1.351 P1, pink), delta (B.1.617.1, green), and omicron SARS-CoV-2 (B.1.1.529, red) compared to wild-type SARS-CoV-2 (Wuhan variant). Black font color indicates mutations shared by several variants, while mutations specific for one variant are indicated by the corresponding font color.

All of these variants have mutations in the RBD with amino acid substitutions at different positions, leading to alpha RBD (N501Y), beta RBD (K417N, E484K, N501Y), gamma RBD (K417T, E484K, N501Y), and delta RBD (later reclassified as kappa RBD: E484Q, L452R) (**Figure 1**) (11). The omicron RBD has at least 15 amino acid substitutions compared to the wt including the positions K417N, E484A, and N501Y known from the other variants and 12 new mutations (G339D, S371L, S373P, S375F, N440K, G446S, S477N, T478K, Q493R, G496S, Q498R, Y505H) (12, 13).

We hypothesized that an Ab recognition of differently mutated RBD versions, i.e., Ab titers determined in ELISA, will most likely correlate well to the neutralizing potential of these Abs against a systemic infection with the corresponding SARS-CoV-2 variants. We initiated this study in 2021, analyzing both total and neutralizing IgG Ab titers in serum samples from people vaccinated with mRNA vaccines BNT162b2 (Tozinameran, Comirnaty, Biontech/Pfizer) and mRNA-1273 (Elasomeran, Moderna) against wt SARS-CoV-2, i.e., sequences used for the development of the first generation of vaccines, and extended it to further globally spreading SARS-CoV-2 VOCs.

## Materials and Methods

Reagents were obtained from the following manufacturers: Advansta Corporation (San Jose, USA): WesternBright Sirius^®^; Carl Roth GmbH & Co. KG (Karlsruhe, Germany): ROTI^®^Stock 10× PBS, ROTI^®^Stock 10× PBS-T, sodium chloride (≥99.5%), sodium dodecyl sulfate (SDS; ≥99.5%), and sulfuric acid; Promega GmbH (Mannheim, Germany): peroxidase-conjugated anti-human IgG Ab; GenScript (Leiden, Netherlands): SARS-CoV-2 Spike protein RBD (Omicron Variant, His Tag); Seramun Diagnostika GmbH (Heidesee, Germany): TMB substrate solution; SERVA Electrophoresis GmbH (Heidelberg, Germany): acrylamide/bis(acrylamide) (30% T, 2.67% C), BlueBlock PF 10×, Coomassie Brilliant Blue G-250, TEMED, and trypsin (sequencing grade, MS approved); Sigma-Aldrich Chemie GmbH (Taufkirchen, Germany): ExtrAvidin-Peroxidase, imidazole (≥99.5%), 2-Mercaptoethanol (BioUltra), and polyethylenimin (PEI); Sino Biological (Eschborn, Germany): ACE-2 (His-tagged, biotinylated); Surmodics IVD, Inc. (Eden Prairie, USA): StabilZyme™ SELECT Assay diluent (Protein-free); Thermo Fisher Scientific (Waltham, MA, USA): goat anti-human IgA secondary Ab-HRP, Gibco DMEM, Gibco GlutaMAX Supplement, Gibco 100× MEM Non-Essential Amino Acids Solution, penicillin/streptomycin (10,000 U/mL), Gibco Fetal Bovine Serum (FBS), and SuperBlock^®^ (PBS).

### Serum Samples

Serum samples from vaccinated people (**Table S1**) were obtained from the Division of Rheumatology (Department of Internal Medicine, Universität Leipzig; 8 men, 4 women, age 22–57 years, average age 31 years), and plasma samples from vaccinated people were obtained from Adversis Pharma GmbH (4 men, 13 women, age 27–59 years, average age 44 years). SARS-CoV-2 negative serum samples collected before 2015 were obtained from a population-based LIFE-Adult study of the Leipzig Research Center for Civilization Disease (LIFE) (14). These samples have been processed and stored by the team of the Leipzig Medical Biobank.

### Receptor-Binding Domain Protein Expression in HEK293S Cells and Purification

The target sequence obtained from a pUC vector (RBD 318-541bp, GenScript) was cloned into the expression vector pHLsec (15) with a C-terminal His-tag. The vectors of SARS-CoV-2 variants were obtained by mutating the individual amino acids (Quikchange mutagenesis) in the pUC vector to insert the mutation sites for alpha RBD (B.1.1.7: N501Y), beta RBD (B.1.351: K417N, E484K, N501Y), delta RBD (B.1.617.1, later reclassified as kappa RBD: E484Q, L452R), and gamma RBD (B.1.1.28 P.1: K417T, E484K, N501Y). Variant B.1.1.529 (Omicron) RBD was purchased as a purified protein C-terminally elongated with a His-tag (GenScript). For transient transfection of the RBDs into HEK293S cells, plasmid vector (3 mg) and PEI (4.5 mg) were incubated in DMEM (150 mL; 10 mmol/L MEM Non-Essential Amino Acids Solution, 2 mmol/L GlutaMAX) at room temperature (RT). After 15 min, transfection mix (25 mL) and penicillin/streptomycin (10,000 U/mL) solution (2.5 mL) diluted in DMEM (225 mL; 10% FBS, 10 mmol/L MEM Non-Essential Amino Acids Solution, 2 mmol/L) were added to the HEK293S cells in each RollerBottle (Greiner Bio-One GmbH, Frickenhausen, Germany) and incubated at 37°C for 5 days. The medium was harvested, and the protein was purified *via* immobilized metal ion affinity chromatography using a HisTrap™ HP (GE Healthcare, Chicago, IL, USA) column followed by size-exclusion chromatography using a HiLoad™ 16/600 Superdex™ 200 pg column (GE Healthcare, Chicago, IL, USA) following the manufacturer’s guidelines.

### Enzyme-Linked Immunosorbent Assay

Medium binding microplates (Greiner Bio-One; 12xF8, PS, F-bottom) were coated with SARS-CoV-2 RBD protein (75 ng/well) in PBS containing NaCl (0.1 mol/L) at 4°C overnight. Wells were washed three times with PBS-T (300 µL/well) and blocked with Superblock (200 µl/well) at RT for 1 h. Equal volumes of 17 serum samples collected from patients with PCR-confirmed SARS-CoV-2 infections and 192 serum samples collected before 2015 were mixed to obtain positive and negative control pools, respectively. Control pools and serum samples were diluted 100-fold in Assay diluent, added to the plate (100 µl/well), and incubated at RT for 45 min. Wells were washed three times with PBS-T (300 µl/well), anti-human IgG-HRP (100 µl/well; diluted 25,000-fold in Stabilzyme) was added, and incubated at RT. After 30 min, the solution was removed, wells were washed three times with PBS-T (300 µl/well), and TMB substrate solution was added (100 µl/well). After 30 min, the reaction was stopped by the addition of sulfuric acid (0.5 mol/l; 100 µl/well) and the absorbance (OD) was recorded at 450 nm using a microplate reader (SpectraMax Paradigm, Molecular Devices, München, Germany). All serum samples collected 2 weeks after the third vaccination were additionally tested using a 500-fold dilution in assay diluent.

### Angiotensin-Converting Enzyme-2 Inhibition Assay

Medium-binding microplates (Greiner Bio-One; 12xF8, PS, F-bottom) were coated with 37.5 ng SARS-CoV-2 RBD protein per well in PBS containing NaCl (0.1 mol/L) at 4°C overnight. The blocking was performed as described for ELISA. Serum samples were diluted 10-fold in assay diluent and incubated (RT, 45 min). Wells were washed three times with PBS-T (300 µl/well). An ACE-2 solution (100 µl 1 mmol/L; 100 ng/well ACE-2) was added and incubated (37°C, 45 min). Wells were washed three times with PBS-T (300 µl/well). ExtrAvidin-Peroxidase (100 µl/well biotin-avidin, diluted 4,000-fold in Stabilzyme) was added, and the plate was incubated (RT, 30 min). Subsequent washing steps and development were identical to the ELISA protocol described above. Control wells were treated in the same way as serum samples, but without the addition of either serum (negative control, 0% inhibition) or ACE-2 receptor (positive control, 100% inhibition). The data were normalized to negative controls (no serum): Neutralization (%) = 100× (1 - [OD_450_ of sample/OD_450_ of negative control]) (16).

### Statistical Analysis

Graphs were created with GraphPad Prism 5.02 (GraphPad Software, Inc., San Diego, CA, USA) and Origin Pro 8G (Friedrichsdorf/Ts, Germany). Statistical analyses were performed with GraphPad Prism. Box plots were created using Whiskers: Min to Max with 95% CI of the mean, and the significant differences were checked by one-way ANOVA (P < 0.05) and Tukey’s multiple comparison test. Linear regression was used to calculate P and R^2^ values.

## Results

**Figure 2:**
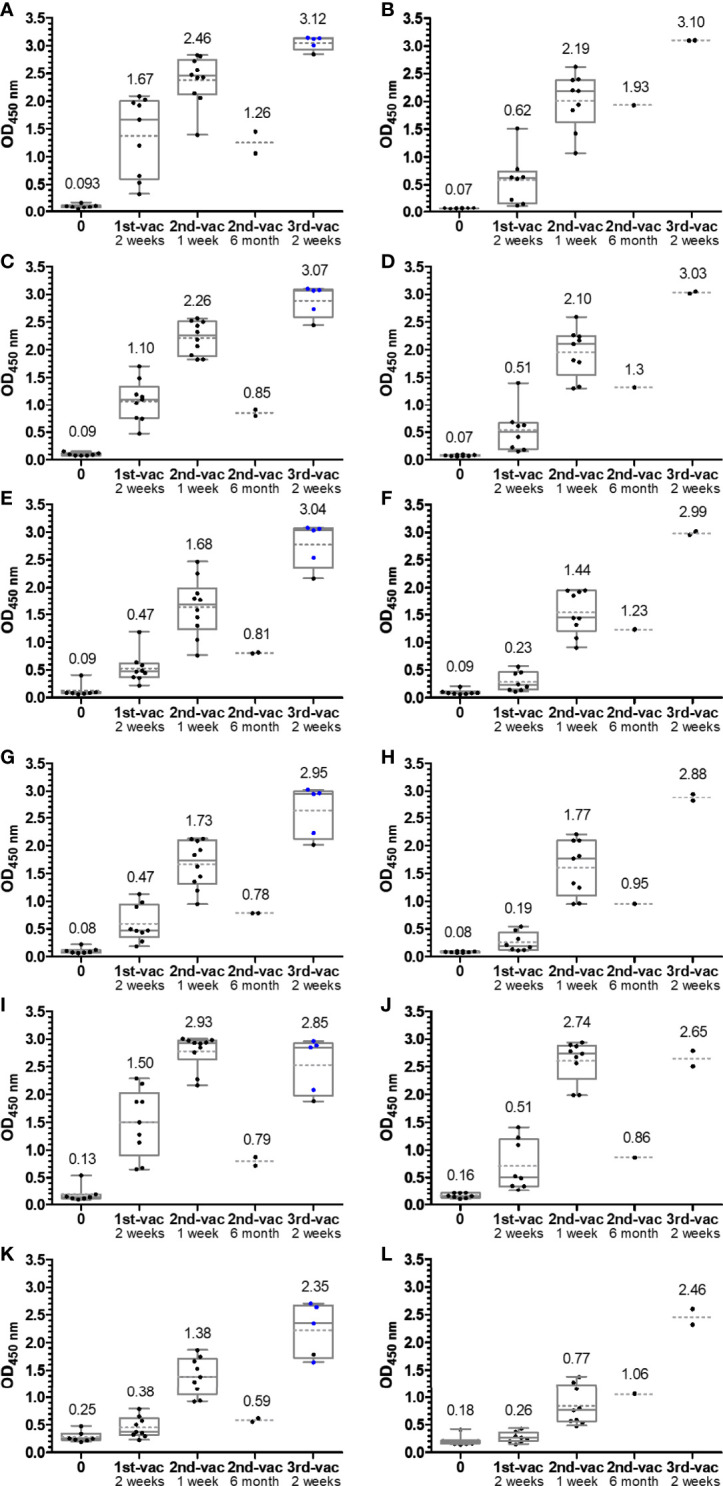
Box plots of the OD_450_ values obtained in an anti-RBD IgG ELISA using wild-type **(A, B)**, alpha **(C, D)**, beta **(E, F)**, gamma **(G, H)**, delta **(I, J)**, and omicron RBDs **(K, L)**. Serum or plasma samples were obtained from people vaccinated with mRNA-1273 (left panels **A, C, E, G, I, K**) or BNT162b2 (right panels **B, D, F, H, J, L**). Sera collected from people with heterologous booster vaccinations (2× mRNA-1273 and once BNT162b2) are indicated as blue dots. The median values are provided above the boxes and are additionally indicated as gray horizontal lines in the boxes, while the mean values are shown as gray horizontal dotted lines.

**Figure 3:**
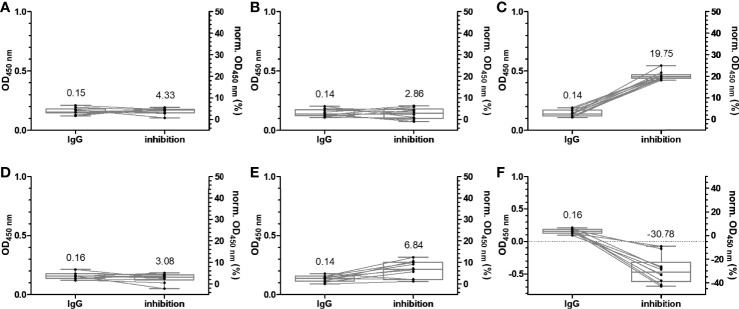
Box plots of the OD_450_ values obtained in an anti-RBD IgG ELISA and normalized OD_450_ values of the inhibition assay using wild-type **(A)**, alpha **(B)**, beta **(C)**, gamma **(D)**, delta **(E)**, and omicron RBDs **(F)**. Serum and plasma samples were collected from 20 patients in the year 2015, which supposedly were not exposed to SARS-CoV-2 or SARS-CoV-1. The relationships of all samples between the two assays are indicated by lines of association. The median values are provided above the boxes and are additionally indicated as horizontal gray lines inside the boxes.

The RBD proteins expressed in-house and the commercial omicron RBD, which all carried a C-terminal His-tag, were highly pure according to Sodium Dodecylsulfate Polyacrylamide Gel Electrophoresis (SDS-PAGE) always showing a dominant band at an apparent molecular weight of ~32 kDa and were also recognized by anti-His IgG Ab (**Figure S1**). The protein identity was confirmed by tandem mass spectrometry after an in-gel digest (**Figure S2**). When the binding of the RBDs to the microtiter plate was probed in a dilution series using the same anti-His-tag IgG Ab that recognized all RBDs in the immunoblot, the wt RBD showed a clear concentration-dependent sigmoidal increase (**Figure S3**). Considering the conflict between sensitivity and assay costs, i.e., high vs. low coating quantities of RBD, we always coated the plates with 75 ng of wt RBD per well, which corresponded to an OD value of ~90% of the plateau observed in the dilution series. The dilution series of the mutated RBDs indicated very similar coating properties as the wt RBD, except for alpha RBD. This curve was shifted around 2-fold, indicating a worse coating. However, this minor loss in sensitivity was acceptable, and thus all ELISA relied on the same RBD quantity of 75 ng/well. Serum samples (**Table S1**) were collected shortly before and 2 weeks after the first vaccination, 1 week after the second vaccination, and 2 weeks after the third vaccination. All samples collected before the first vaccination (**Figure 2**) and serum samples collected before 2015 (**Figure 3**) tested negative (OD <0.2) for all six RBDs included in this study. Both mRNA-1273 and BNT162b2 vaccines triggered a strong immune response against wt RBD, even after the first administration, which further increased after the second and third vaccinations (**Figures 2A, B**). Generally, the OD values were always higher for mRNA-1273 than for BNT162b2, as indicated by the median OD values of 0.62 after the first vaccination and 2.19 after the second vaccination compared to those of mRNA-1273, reaching OD values of 1.67 and 2.46, respectively. Since serum and plasma samples were tested, the correlation between both sample types was investigated in the described assay. The standard deviation ranged from 1.0% to 7.4% (one sample 23%), indicating a very good correlation, confirming that the assay provides comparable results for serum and plasma samples taken from one person (**Figure S4**). The third vaccination increased the OD values to ~3.1 for both vaccines. However, it should be noted that in the case of mRNA-1273, cross-inoculations are present and only one sample received mRNA-1273 as a third inoculation.

**Figure 4:**
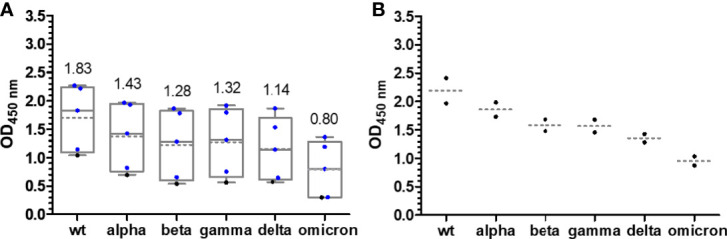
Box plots of the OD_450_ values obtained in an anti-RBD IgG ELISA for people vaccinated with mRNA-1273 **(A)** and BNT162b2 **(B)** around 2 weeks after the third vaccination. Serum and plasma samples were diluted 500-fold. Sera collected from people with heterologous booster vaccinations (2× mRNA-1273 and once BNT162b2) are indicated as blue dots. The median values are provided above the boxes and are additionally indicated as gray horizontal lines in the boxes, while the mean values are shown as gray horizontal dotted lines.

The median OD values for the alpha variant and the distribution were similar to those of wt RBD (**Figures 2C, D****)**, although differences were observed for individual samples indicating that sera contained IgG Abs recognizing the RBD around mutation site N501Y. The additional mutations present in beta RBD (E484K and K417N) and gamma RBD (E484K and K417T) reduced the median OD values to 0.47 after the first and to ~1.7 after the second vaccination with mRNA-1273 and to a similar degree to ~0.2 and ~1.6 for BNT162b2 (**Figures 2E–H**). The third vaccination increased the OD values to ~3. Interestingly, the delta RBD was recognized better than the beta and gamma RBDs and almost as well as the wt RBD after the first vaccination and even better for both vaccines after the second vaccination (**Figures 2I, J****)**. This might be related to the N501Y mutation, which significantly reduced the recognition of alpha RBD but is missing in delta RBD. Additionally, mutation E484Q in delta RBD might disturb Ab binding less than E484K due to its reversed charge. Unexpectedly, the third vaccination did not increase the OD values but resulted in slightly lower median OD values for both vaccines. Despite a low effect, it may indicate that several vaccinations with wt RBD might select IgG Abs recognizing this RBD the best, while less specific or weaker binding IgG Abs that recognize mutated RBDs, such as delta RBD, are lost over time. The omicron RBD variant carrying 15 mutations including the mutation sites at 417, 484, and 501, present in alpha, beta, and gamma RBDs, yielded the lowest median OD values of 0.38 (0.26) after the first, 1.38 (0.77) after the second, and only 2.35 (1.06) after the third vaccination with mRNA-1273 (BNT162b2) (**Figures 2K, L****)**. Thus, the third vaccination strongly increased the OD values, especially for BNT162b2 where the number tripled. Besides the time points shortly after each vaccination, serum samples from a few people were collected 6 months after the second vaccination and before the third vaccination. The exact course for both vaccines of some patients is shown in the supplement (**Figures S5, S6**). Despite the low sample numbers, there was a clear trend that the Ab titers decreased significantly for both vaccines and all RBDs (**Figure 2**). Interestingly, the Ab titers in the BNT162b2 group decreased only slightly, while the titers in the mRNA-1273 group decreased to the levels after the first vaccination and for some RBD variants even lower. Importantly, both vaccines induced Abs that recognized all mutated RBDs with increasing titers after each vaccination. Due to the high OD values obtained after the second and third vaccination, the sera were measured again at a higher dilution, i.e., 500-fold (200-fold) instead of 100-fold (**Figure 4**; **Figures S7, S8**). Remarkably, the median OD values of the BNT162b2 group were always higher than those for the mRNA-1273 group, for example, 2.19 vs. 1.83 for wt RBD and 0.95 vs. 0.80 for omicron RBD after the third vaccination (**Figure 4**). Generally, the wt RBD was recognized the best, followed by alpha, beta, gamma, and delta RBDs with intermediate values, while the lowest OD values were observed for omicron RBD. Importantly, all tested sera showed high OD values against all RBDs, indicating a good immunological protection against systemic infections. All serum samples except one also contained low titers of anti-RBD IgA Abs, which might prevent infections shortly after vaccination, but this was not further investigated (**Figure S9**).

More appealing was the question whether anti-RBD IgG titers can be used to predict neutralization. Neutralization of sera is typically studied using either live SARS-CoV-2 and Vero cell lines naturally expressing the ACE-2-receptor [conventional virus neutralization test (cVNT)] or a pseudovirus-based virus neutralization test (pVNT) using for example an adenovirus and a HEK293T cell line engineered to express spike protein and ACE-2 receptor, respectively. The neutralization effect is measured by the number of lysed cells in the presence of inactivated serum presumed to have no neutralizing antibodies (nAbs) (control) and serum or plasma to be tested for nAbs, which would rescue the cell line. Although these assays are well established and the SARS-CoV-2/Vero assay is considered as the “gold standard” for studying neutralization of sera, they are time-consuming, are expensive, and require well-equipped laboratories and well-trained personnel (Biosafety Level 3 when using live SARS-CoV-2). This typically limits their application to small sample numbers as part of research projects and prevents an upscaling for community testing. Thus, we relied on a recently reported SARS-CoV-2 surrogate virus neutralization test (sVNT) probing the protein–protein interaction between RBD and ACE-2, which represents the first step of SARS-CoV-2 infections. The sVNT was demonstrated to predict neutralizaion of sera, while showing a better correlation with the cVNT than the pVNT (17, 18). We prefer the term inhibition, as the RBD-ACE-2 binding is inhibited by Abs or other serum components, instead of neutralization, which typically refers to the results of a cell-based assay. Although a strong correlation between both assay types was shown, it remains open how inhibition determined by sVNT correlates to neutralization determined by cVNT or pVNT. It should be noted that both neutralization and inhibition are terms used in *in vitro* assays that allow predicting reduced infection rates of SARS-CoV-2 in a host, although a direct correlation is not possible.

**Figure 5:**
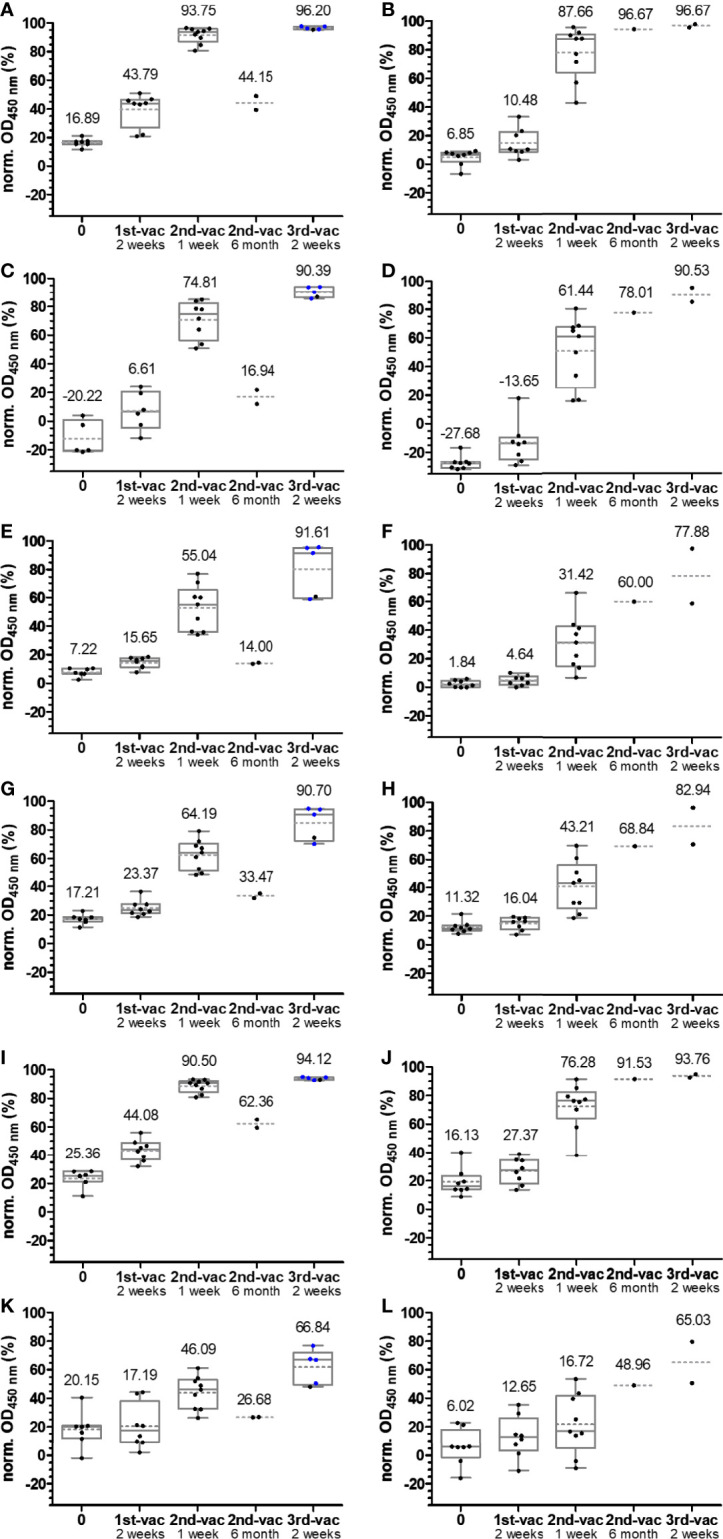
Box plots of the normalized OD_450_ values obtained in the inhibition assay using wild-type **(A, B)**, alpha **(C, D)**, beta **(E, F)**, gamma **(G, H)**, delta **(I, J)**, and omicron RBDs **(K, L)**. Serum or plasma samples were obtained from people vaccinated with mRNA-1273 (left panels **A, C, E, G, I, K**) or BNT162b2 (right panels **B, D, F, H, J, L**). Sera collected from people with heterologous booster vaccinations (2× mRNA-1273 and once BNT162b2) are indicated as blue dots. The median values are provided above the boxes and are additionally indicated as gray horizontal lines in the boxes, while the mean values are shown as gray horizontal dotted lines.

Another important criterion is the prerequisite of cell cultures to use inactivated serum (19), while we intended to use untreated serum to consider possible interferences from serum components in their native form, which may elevate or inhibit the neutralizing effects of Abs. The assay design was based on the IgG-ELISA, i.e., coating an RBD (37.5 ng/well) followed by the addition of serum. This should allow a good comparability between IgG recognition and ACE-2 inhibition for each RBD and among different RBDs considering the very similar coating properties of all six RBDs, as discussed above. The protein–protein interaction was probed by adding biotinylated ACE-2, which was detected with HRP-conjugated ExtrAvidin. IgG Abs recognizing the RBD would be considered inhibitory, if they reduce or prevent binding of ACE-2 in the next step, allowing to scale inhibition from 0%, i.e., samples containing no nAbs (no serum), to 100% for a complete neutralization (no ACE-2 added). A serial dilution of ACE-2 showed a concentration-dependent increase of the OD values for all RBDs, reaching plateaus at OD values of ~1.5 (omicron RBD) to ~3.0 (wt RBD), indicating a saturation above 500 ng ACE-2 per well (**Figure S10**). Considering the assay costs, an ACE-2 concentration of 100 ng/well was used afterward, which provided OD values in the dynamic range of the assay ranging from ~1.0 (omicron RBD) to 2.5 (wt RBD). For a better comparison with the previously described IgG ELISA, the serum dilution of 1:100 was also tested in the ACE-2 inhibition assay, which showed no inhibitory effect for the serum samples (**Figure S11**). When a 10-fold dilution was tested, the inhibitory effect was already observed for serum samples collected 1 week after the second vaccination (**Figure S11**). This dilution was used for all of the following experiments. Expectedly, the wt RBD–ACE-2 interaction was not disturbed by sera collected before 2015, as indicated by the median inhibition of 4.3% (**Figure 3A**). Similar median inhibitions were observed for alpha, gamma, and delta RBDs (**Figures 3B, D, E****)**, while higher values (19.75%) were obtained for beta RBD and lower values (-30.78%) for omicron RBD (**Figures 3C, F****)**. A similar inhibition range was obtained for all samples collected before the first vaccination, i.e., from -20.22% to 25.36% (**Figure 5**; **Figure S12**), which fits well to previously reported data for wt RBD using sample sets from Singapore and China (17). Thus, values above 30% can be considered as inhibitory. The median inhibition of samples of the mRNA-1273 group increased from initially 16.89% to 43.79%, 93.75%, and 96.20% after the first, second, and third vaccination, indicating increasing strong inhibitory effects for wt RBD in all people already after the second vaccination (**Figures 5A, B****)**. As expected from the lower IgG titers observed for BNT162b2 compared to mRNA-1273, the median inhibition increased slightly after the vaccination but remained below the cutoff. A good correlation between serum and plasma samples taken from the same individual was also observed for this assay with standard deviations between 0.2% and 19.2% (**Figure S4**). The median inhibition strongly increased after the second vaccination, but there was a large difference between the worst and best results (40% to 90%), while the third vaccination induced a very strong inhibition of 96.67% in all people, which was very similar to the mRNA-1273 group (**Figures 5A, B****)**. Thus, a good correlation between the IgG titers and the inhibition assay was observed (**Figure S13**). Similar trends were observed for the other RBDs, although the inhibition was always weaker, reaching high levels of ~90% only after the third vaccination (**Figures 5C–J**), except for omicron RBD that showed an inhibition of only ~66% for both vaccines (**Figures 5K, L****)**. Generally, the median inhibition was slightly stronger for the mRNA-1273 vaccine than that for the BNT162b2 vaccine, especially at the earlier time points, but was lower in the mRNA-1273 group than that in the BNT162b2 group shortly before the third vaccination (around 6 months after the first vaccination). Importantly, the IgG-ELISA and the sVNT correlated well, indicating that both vaccines lead to high IgG titers strongly inhibiting RBD–ACE-2 interactions and thus most likely strong neutralizing effects with the promise of a much milder course of COVID-19 for all six tested SARS-CoV-2 variants.

## Discussion

Both mRNA-based vaccines studied here initiated a strong immune response, indicated by the anti-wt-RBD IgG ELISA, which recognizes the SARS-CoV-2 S-protein. People vaccinated with BNT162b2 generally had slightly lower Ab titers after the second vaccination than people vaccinated with mRNA-1273. The third vaccination pushed the titers well above the previous values, independent of the vaccine or vaccine combinations (2× mRNA-1273 + BNT162b2), leading to similar titers. However, comparisons between the BNT162b2 and mRNA-1273 cohorts have to be evaluated with caution due to the small sample sets and as the groups were not matched for age and sex, which may affect the serum titers. The weaker recognition of the alpha RBD compared to wt RBD and the weaker inhibition of RBD–ACE-2 interaction identified the region around Asn-501 as a dominant immunogenic sequence. Alternatively, mutation N501Y could also alter the RBD structure and thereby influence recognition of other epitopes. The importance of this escape mutation is further supported by beta, gamma, and omicron variants of SARS-CoV-2, which all contain the N501Y mutation site (**Figure 1**). Importantly, structural data show that Tyr-501 is inserted in a cavity at the RBD-binding site of ACE-2, most likely increasing the cellular uptake of SARS-CoV-2 (18, 20–24). The weaker recognition of alpha and beta variants in an IgG ELISA was also observed by Lu et al. (25) and Chen et al. (26). Further decreased recognition and inhibition of beta and gamma RBDs point to the additional mutations of residues Lys-417 and Glu-484 (**Figure 1**) that might also be parts of immunogenic sequences, as shown for E484K in an extended alpha variant (27). The importance of Asn-501 mutations to escape the immune system of vaccinated people is also supported by the good recognition and strong inhibition of ACE-2 binding for the delta variant, which lacks this mutation. The good recognition of the delta variant further shows that mutation E484Q has a much weaker effect on IgG binding than mutation E484K, indicating that a neutral residue is tolerated (most likely also the E484A mutation in the omicron variant), while a large positively charged side chain disturbs IgG binding. Expectedly, the omicron variant with mutations K417N, E484A, and N501Y affecting the Ab recognition, as discussed above, and the additional 12 mutations distributed between G339D and Y505H showed the lowest OD values in all cases, as previously reported (12, 20). However, IgG Ab titers increased 3-fold after the third vaccination, providing a moderate neutralization effect of ~60%. The few progression samples collected around 6 months after the second and before the third vaccination showed the expected decrease in titers (28–31), which appeared to be more prevalent with mRNA-1273, although the low sample numbers do not allow valid conclusions.

Simple and rapid SARS-CoV-2 surrogate virus inhibition assays were successfully established for different RBDs and the ACE-2 receptor protein. Most importantly, the coating of all six RBDs was equally efficient, as indicated by an anti-His-tag Ab, which should allow a reliable comparison of ACE-2 binding to coated RBDs and judging the neutralizing effect of anti-RBD Abs present in serum and plasma samples. This assay will allow a simple screening of SARS-CoV-2 virus neutralization in regular laboratories equipped for ELISA without the need for Biosafety Level 3 laboratories. Additionally, it is much faster and cheaper than cVNTs and pVNTs, can be easily upscaled, does not require inactivation of serum samples, and can be applied to both serum and plasma samples. This confirms the benefits originally reported by Tan et al. (17) sVNTs, although we reversed the assay by coating an RBD to the plate and testing the binding of ACE-2 present in solution or spiked to serum and plasma samples. Furthermore, S-protein-negative samples showed a similar distribution as previously reported (17) and a good linear correlation between increasing IgG Ab levels and inhibition efficiency. Thus, an initially stronger inhibition takes place in the mRNA-1273 samples, which reaches similar levels after the third vaccination with both vaccines, i.e., an equally strong inhibition of wt RBD binding (~96.6%) (32). A lower inhibition of alpha, beta, and gamma RBDs after two vaccinations is consistent with previous assumptions that alpha, beta, and gamma SARS-CoV-2 variants are neutralized at a lower degree in individuals vaccinated with Ad26.COV2.S using a pVNT assay (23). Interestingly, samples collected after the third vaccination strongly inhibited the alpha, beta, gamma, and delta variants (>90%), almost reaching the level obtained for wt RBD (33–35). This clearly shows that these VOCs do not escape nAbs circulating in people vaccinated three times with mRNA vaccines, confirming previous assumptions about the alpha variant (36, 37). This is in partial contrast to a previous report indicating that the delta variant can escape nAbs in vaccinated people based on data obtained in an S-protein-based pVNT (38). Abs that might be directed against other regions of the S-protein, such as the N-terminal domain (NTD) in the S1-region, may also play an important role in reducing the inhibition of IgG Abs (39), which would not be detected in our RBD-based assay. Therefore, it would be interesting to study the entire spike protein in the established assay, in addition to any new VOCs of RBD that may emerge in the future. Such an assay would also evaluate cryptic epitopes regulating the so-called “up” and “down” conformation of the RBD that indirectly control RBD–ACE-2 binding (40). Since all currently available vaccines including the two mRNA-based vaccines tested here rely on the wt sequence of the original SARS-CoV-2 strain identified in Wuhan, it was expected that this evolutionary pressure on the virus would trigger mutations to escape vaccine-based nAbs, as exemplified by the omicron variant, as demonstrated in recent studies (32, 41). However, a fairly good inhibition of ~66% was achieved here after the third vaccination, which should protect people from a severe etiopathology. This is in agreement with data obtained in a modified virus-like particle neutralization test (hiVNT) system based on lentiviruses (42). Importantly, the data presented here show a good correlation between sVNT inhibition values and IgG titers for all tested variants, confirming the strong neutralization effects observed after three vaccinations.

## Conclusion

The occurrence of new SARS-CoV-2 variants containing known mutations of VOCs or new mutations in the RBD of the S-protein always raises concerns about the effectiveness of current vaccines with respect to the produced nAbs. We established a simple sVNT by coating the RBDs of wt, alpha, beta, gamma, delta, and omicron variants of SARS-CoV-2 to a microtiter plate and probing the ACE-2 receptor binding in competition with Abs present in sera from unvaccinated and vaccinated people. This assay uses similar protocols and the same instruments as regular ELISA tests to determine the Ab titers in serum or plasma samples. Thus, it can be established in many laboratories and easily adapted to new SARS-CoV-2 variants for quantifying neutralization of vaccinated or recovered people, which will allow predicting the risk for severe symptoms or higher lethality. Interestingly, the IgG titers linearly correlated to neutralization for all tested RBD variants, indicating that IgG titers measured a few weeks after vaccination provide a good measure for predicting neutralization and thus the content of nAbs.

## Data Availability Statement

The original contributions presented in the study are included in the article/**Supplementary Material**. Further inquiries can be directed to the corresponding author.

## Ethics Statement

Ethical review and approval were not required for the study on human participants in accordance with the local legislation and institutional requirements. The patients/participants provided their written informed consent to participate in this study.

## Author Contributions

MS, AK, and AB developed and optimized the ELISA and inhibition assay. MS, AK, and FP expressed RBD proteins. MS and DV analyzed and characterized recombinant RBD proteins. AK, MS, and RH summarized and interpreted the data. AK, MS, and RH were major contributors in writing the article. All authors have read and corrected previous article versions and agreed to the published version of the article. AK and RH initiated and supervised the project. RH acquired funding.

## Funding

Funding by the European Union, the European Regional Development Fund (ERDF), and the Free State of Saxony (Grant number 100523073 to RH), as well as the Deutsche Forschungsgemeinschaft (DFG, grant number INST 268/387-1 to RH) is gratefully acknowledged. This project was co-financed by tax funds on the basis of the budget passed by the Saxon state parliament. LIFE-Adult is funded by means of the European Union, by the ERDF, and by funds of the Free State of Saxony within the framework of the excellence initiative.

## Conflict of Interest

MS, AK, AB, and RH maintain close research collaborations with Adversis Pharma GmbH (Leipzig) focusing on the development and optimization of SARS-CoV-2 ELISA.

The remaining authors declare that the research was conducted in the absence of any commercial or financial relationships that could be construed as a potential conflict of interest.

Some serum and plasma samples were obtained from Adversis Pharma GmbH.

## Publisher’s Note

All claims expressed in this article are solely those of the authors and do not necessarily represent those of their affiliated organizations, or those of the publisher, the editors and the reviewers. Any product that may be evaluated in this article, or claim that may be made by its manufacturer, is not guaranteed or endorsed by the publisher.
