# Supplementary material for: Cross-Reactivity of IgG Antibodies and Virus Neutralization in mRNA-Vaccinated People Against Wild-Type SARS-CoV-2 and the Five Most Common SARS-CoV-2 Variants of Concern
Source: Front Immunol. 2022 Jun 15;13:915034. doi: 10.3389/fimmu.2022.915034 (PMC9242094; doi:10.3389/fimmu.2022.915034)
Supplement: Supplementary file 1 [file DataSheet_1.docx]

Supplementary Material

**Table of Contents**

Table S1: Clinical parameters of serum and plasma samples 2

Figure S1: SDS-PAGE and immunoblots of SARS-CoV-2 RBD mutants 4

Figure S2: Protein sequence coverages obtained in tandem mass spectrometry 5

Figure S3: Protein coating in the anti-RBD IgG ELISA using an anti-His antibody 6

Figure S4: Assay results of serum and plasma samples collected from one individual 7

Figure S5: anti-RBD IgG ELISA or the inhibition assay (mRNA-1273) 8

Figure S6: anti-RBD IgG ELISA or the inhibition assay (BNT162b2) 9

Figure S7: anti-RBD IgG ELISA after two vaccinations 10

Figure S8: anti-RBD IgG ELISA after three vaccinations 11

Figure S9: Box plots of OD_450_ values obtained in an anti-wt-RBD IgA ELISA 12

Figure S10: Inhibition assay for wt RBD and RBD variants 13

Figure S11: Box plots of inhibition assay for wt RBD and RBD variants 14

Figure S12: Correlation of anti-RBD IgG ELISA and inhibition assay 16

**Table S1**: Clinical parameters of serum and plasma samples collected from 29 individuals included in the current study vaccinated with mRNA-1273 and BNT162b2.

| **Sample** | **Before** | **1^st^ vac.**  **2 weeks** | **2^nd^ vac.**  **1 week** | **2^nd^ vac.**  **6 months** | **3rd vac.**  **2 weeks** | **1^st^ / 2^rd^**  **vaccine** | **3^rd^**  **vaccine** | **Age** | **Sex** |
| --- | --- | --- | --- | --- | --- | --- | --- | --- | --- |
| **m1** | m1.0 | m1.1 | m1.2 | m1.3 | m1.4 | mRNA-1273 | BNT162b2 | 25 | f |
| **m2** | m2.0 | m2.1 | m2.2 | m2.3 | m2.4 | mRNA-1273 | mRNA-1273 | 32 | m |
| **m3** | m3.0 | m3.1 | m3.2 | - | m3.4 | mRNA-1273 | BNT162b2 | 32 | m |
| **m4** | m4.0 | m4.1 | m4.2 | - | - | mRNA-1273 |  | 28 | f |
| **m5** | m5.0 | m5.1 | m5.2 | - | - | mRNA-1273 |  | 28 | m |
| **m6** | - | - | m6.2 | - | - | mRNA-1278 |  | 31 | m |
| **m7** | m7.0 | m7.1 | m7.2 | - | - | mRNA-1273 |  | 27 | f |
| **m8** | - | m8.1 | m8.2 | - | m8.4 | mRNA-1273 | BNT162b2 | 22 | m |
| **m9** | - | m9.1 | m9.2 | - | m9.4 | mRNA-1273 | BNT162b2 | 24 | m |
| **m10** | m10.0 | m10.1 | m10.2 | - | - | mRNA-1273 |  | 36 | m |
|  |  |  |  |  |  |  |  |  |  |
| **b1** | - | b1.1 | b1.2 | b1.3 | b1.4 | BNT162b2 | BNT162b2 | 57 | m |
| **b2** | - | - | - | b2.3 | b2.4 | BNT162b2 | BNT162b2 | 27 | f |
| **b3** | b3.0 | - | b3.2 | - | - | BNT162b2 |  | 53 | f |
| **b4** | b4.0 | b4.1 | b4.2 | - | - | BNT162b2 |  | 51 | m |
| **b5** | b5.0 | b5.1 | b5.2 | - | - | BNT162b2 |  | 59 | f |
| **b6** | b6.0 | b6.1 | b6.2 | - | - | BNT162b2 |  | 55 | m |
| **b7** | b7.0 | b7.1 | b7.2 | - | - | BNT162b2 |  | 27 | f |
| **b8** | b8.0 | b8.1 | b8.2 | - | - | BNT162b2 |  | 30 | f |
| **b9** | b9.0 | b9.1 | b9.2 | - | - | BNT162b2 |  | 49 | f |
| **b10** | b10.0 | b10.1 | b10.2 | - | - | BNT162b2 |  | 32 | m |
| **b11*** | - | - | - | - | - | BNT162b2 |  | 46 | f |
| **b12*** | - | - | - | - | - | BNT162b2 |  | 37 | w |
| **b13*** | - | - | - | - | - | BNT162b2 |  | 43 | w |
| **b14*** | - | - | - | - | - | BNT162b2 |  | 50 | w |
| **b15*** | - | - | - | - | - | BNT162b2 |  | 42 | w |
| **b16*** | - | - | - | - | - | BNT162b2 |  | 36 | m |
| **b17*** | - | - | - | - | - | BNT162b2 |  | 39 | w |
| **b18*** | - | - | - | - | - | BNT162b2 |  | 49 | w |
| **b19*** | - | - | - | - | - | BNT162b2 |  | 46 | w |

* denotes samples collected at different time points after the second vaccination.


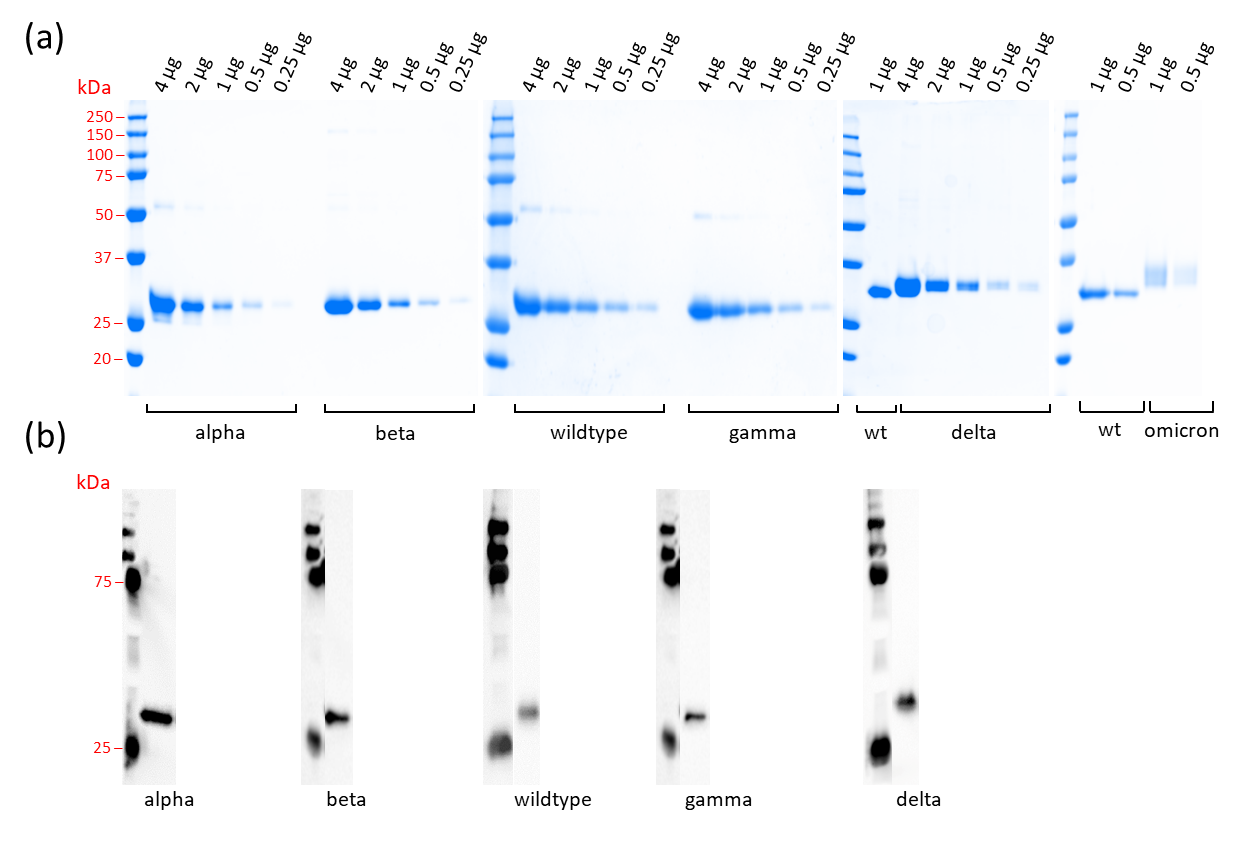


**Figure S1**: SDS-PAGE (a) and corresponding immunoblots (b) of different SARS-CoV-2 RBD mutants loaded at the indicated quantities (32 kDa). (a) Gels were stained with Coomassie Brilliant Blue G-250. Immunoblots were probed with an anti-His IgG Ab as primary antibody (b). The first lane contained marker proteins with the molecular masses indicated in kDa.


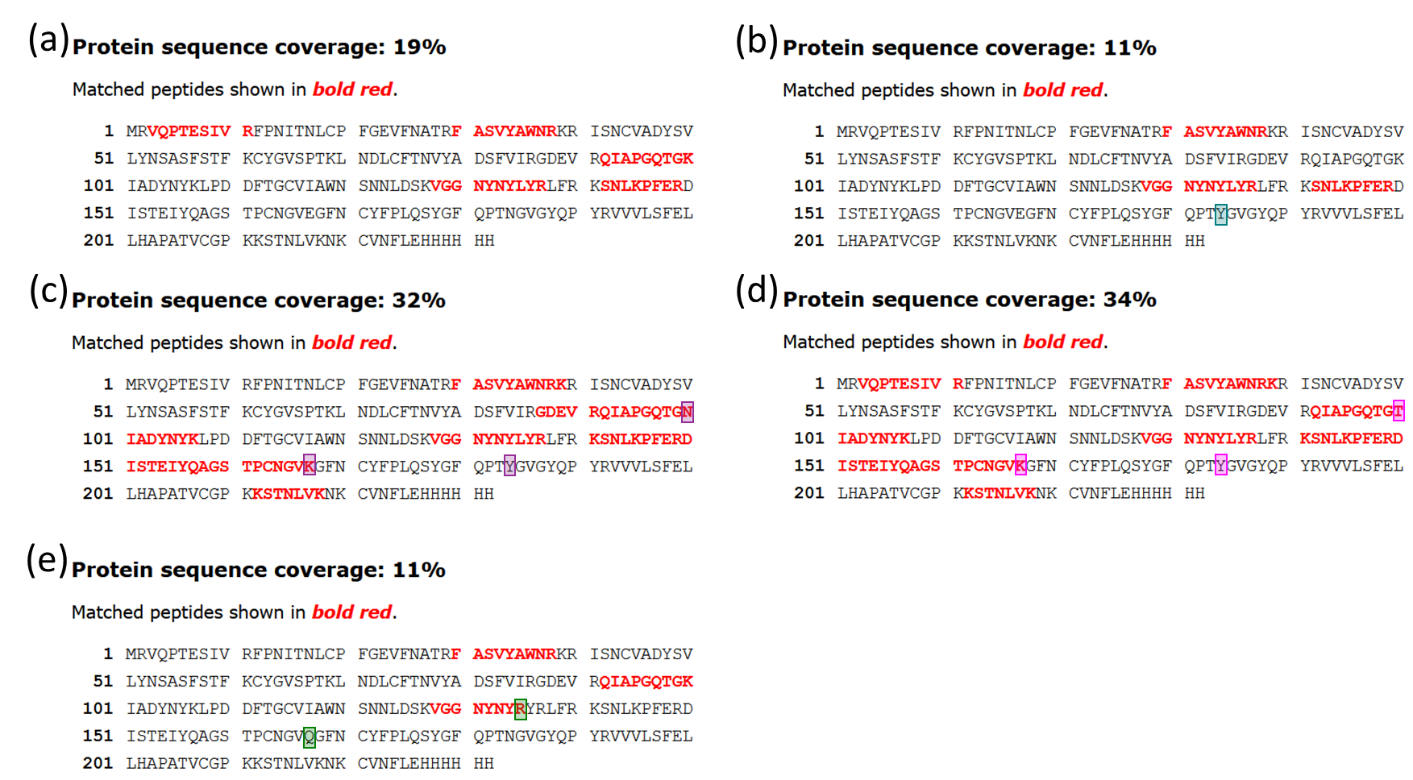


**Figure S2**: Protein sequence coverages obtained in tandem mass spectrometry after in-gel tryptic digestion of in-house wild type (a), alpha (b), beta (c), gamma (d) and delta RBDs (e) separated by SDS-PAGE (Fig. S1). The identified sequences are indicated in red, the mutated residues relative to the wild type sequence are highlighted by the boxes.


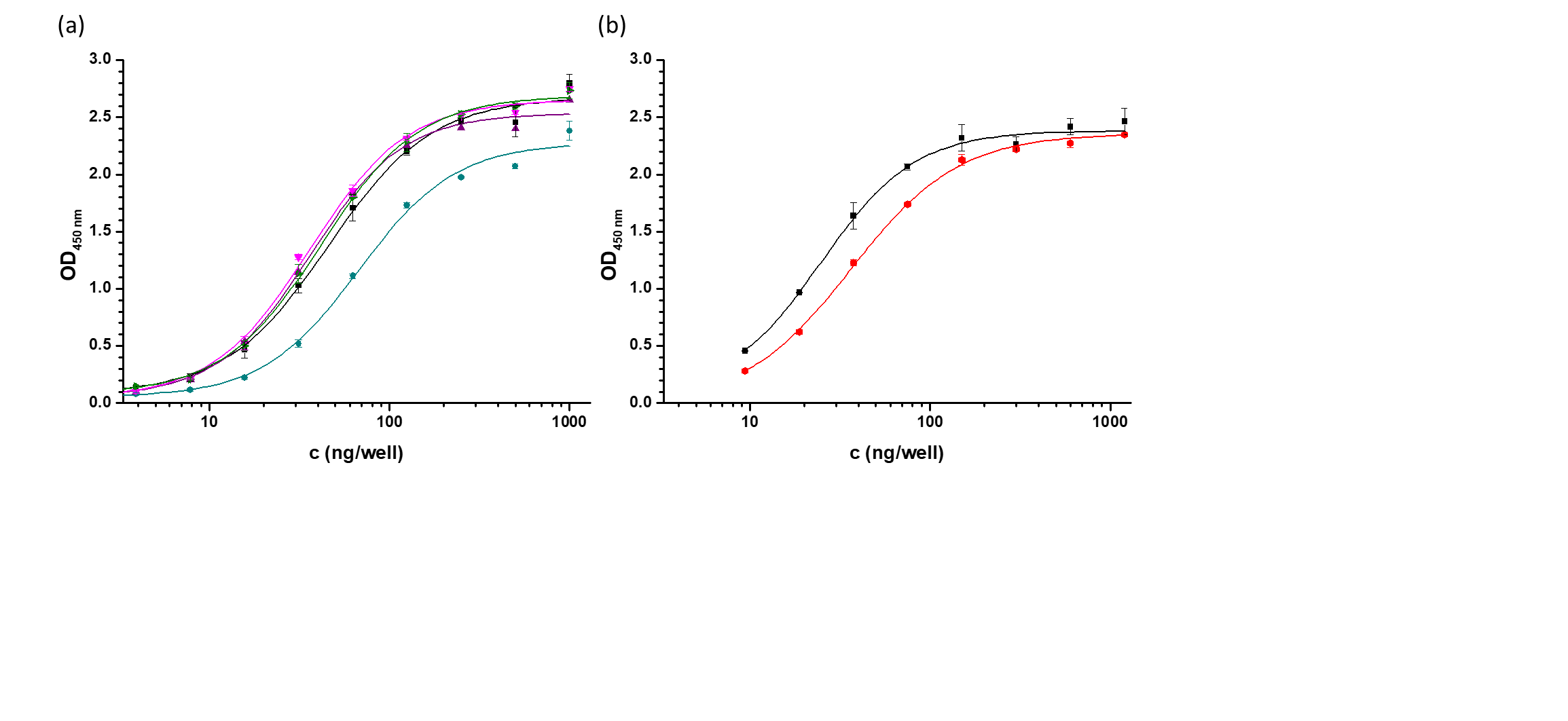


**Figure S3**: OD_450_ values obtained in the anti-RBD IgG ELISA using an anti-His antibody for a dilution series of wild type (black, square), alpha (blue, circle), beta (violet, triangle down), gamma (magenta, triangle up), and delta RBDs (green, triangle right) expressed in-house (a) and wild type (black, square) and commercial omicron RBDs (red, hexagon) (b).


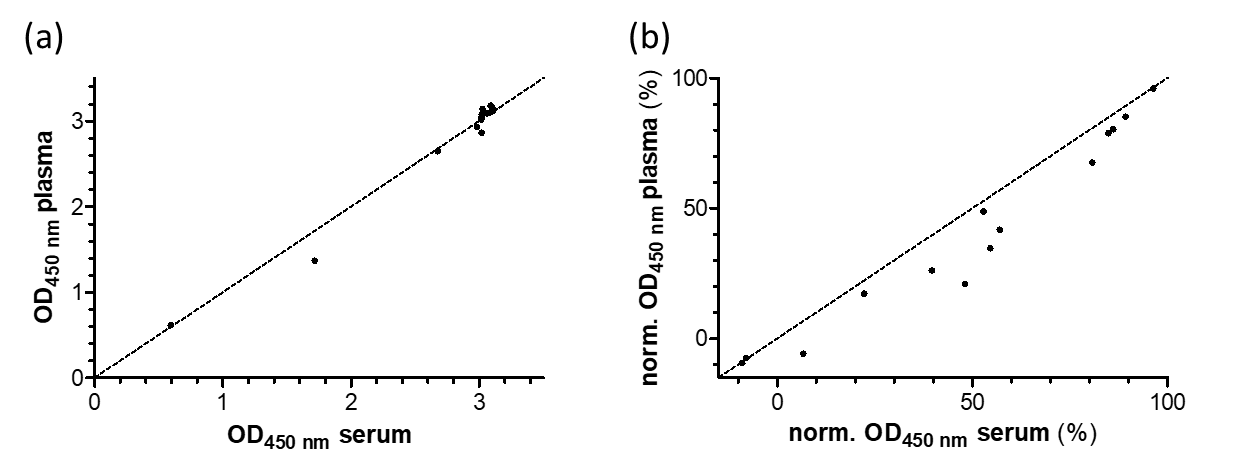


**Figure S4**: Correlation of the OD_450_ values in IgG ELISA (a) and inhibition assay (b) determined for serum and plasma samples collected from the same individual. The dotted line indicates the theoretically expected correlation.


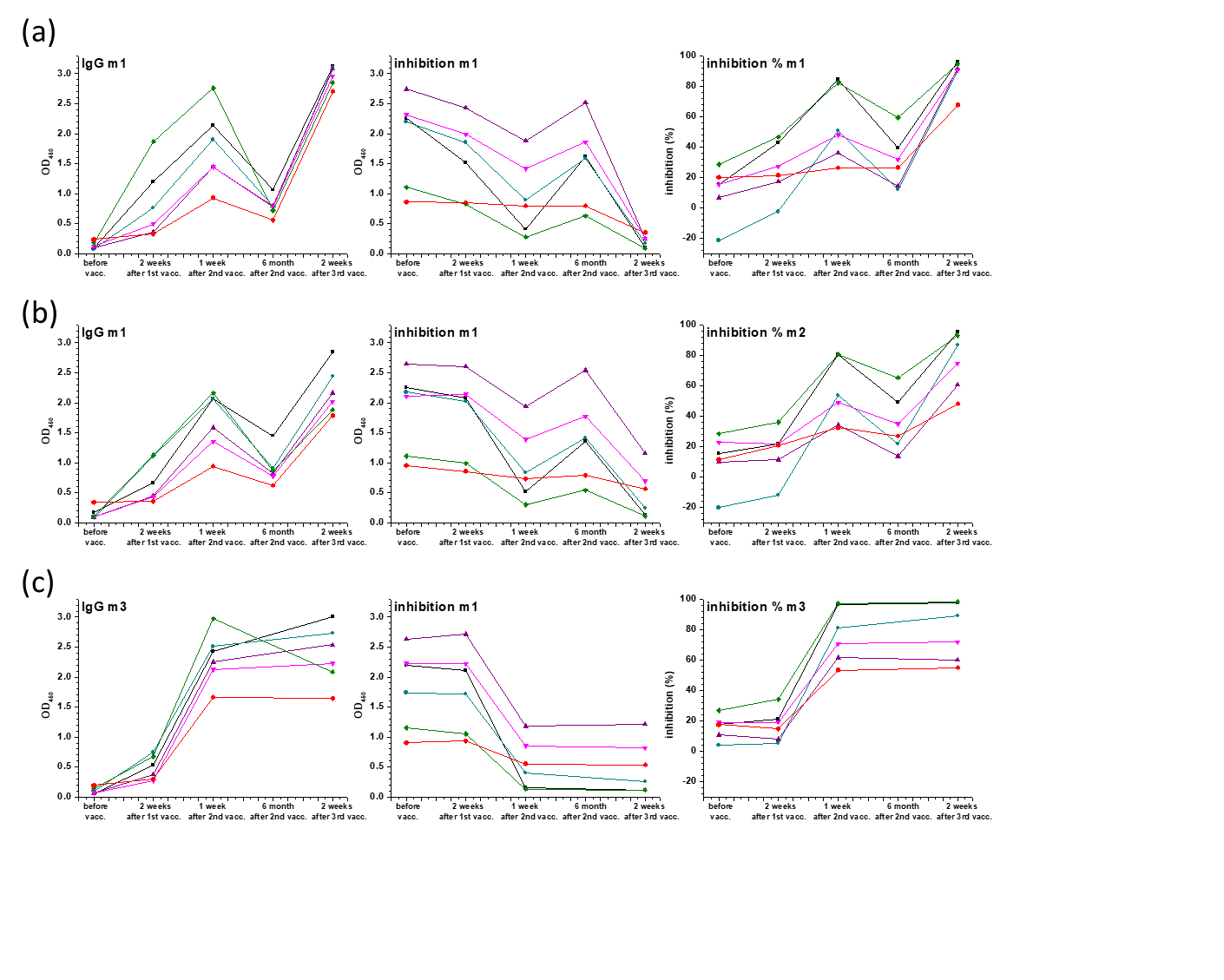


**Figure S5**: OD_450_ values obtained in the anti-RBD IgG ELISA (left) or the inhibition assay (middle) and normalized inhibition values (right) for persons vaccinated m1 (a), m2 (b), and m3 (c) with mRNA-1273. The sera were tested against wild type (black, square), alpha (blue, circle), beta (violet, triangle up), gamma (magenta, triangle down), delta (green, triangle right) and omicron RBDs (red, hexagonal).


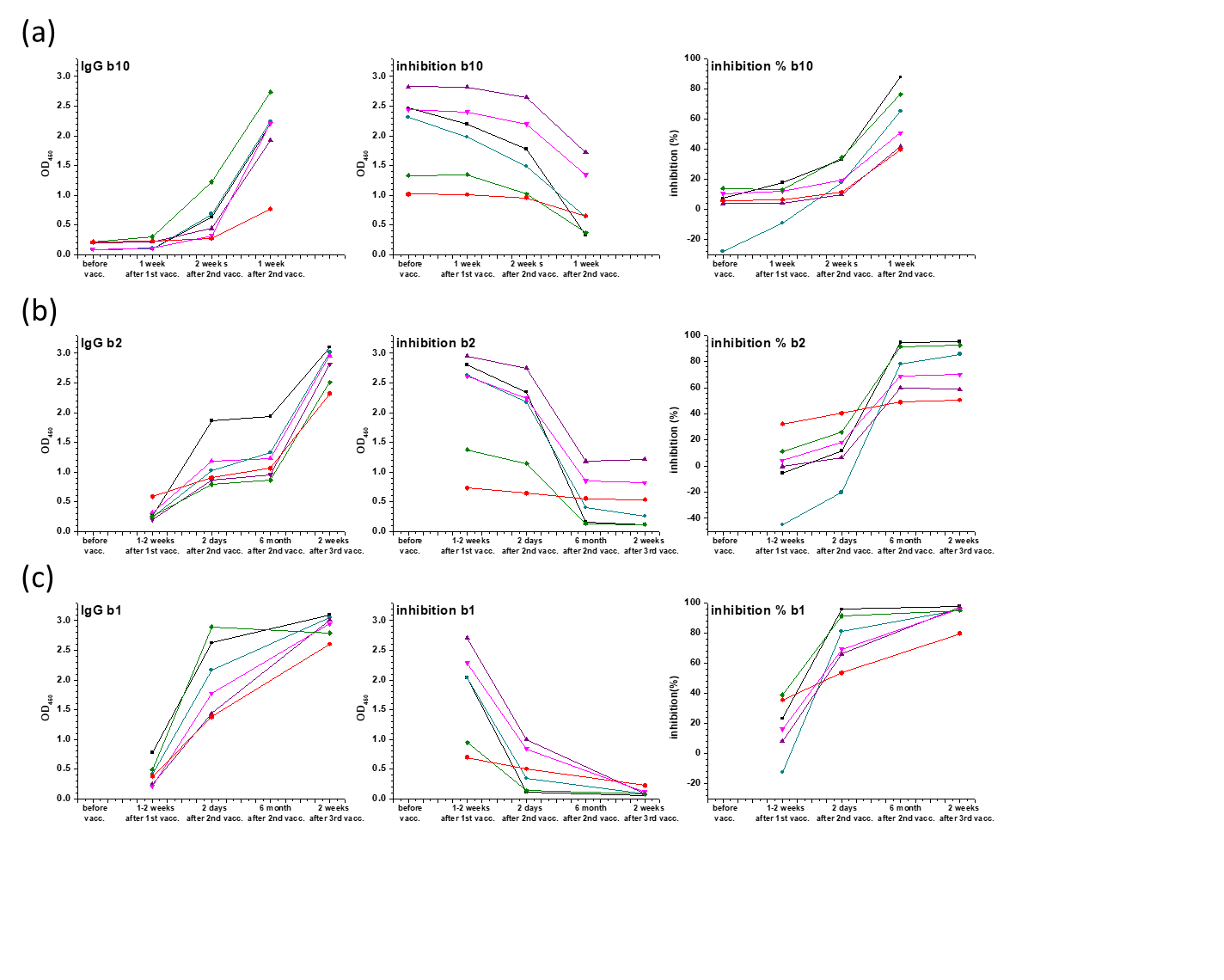


**Figure S6**: OD_450_ values obtained in the anti-RBD IgG ELISA (left) or the inhibition assay (middle) and normalized inhibition values (right) for persons vaccinated m1 (a), m2 (b), and m3 (C) with BNT162b2. Sera were tested against wild type (black, square), alpha (blue, circle), beta (violet, triangle up), gamma (magenta, triangle down), delta (green, triangle right), and omicron RBDs (red, hexagonal).


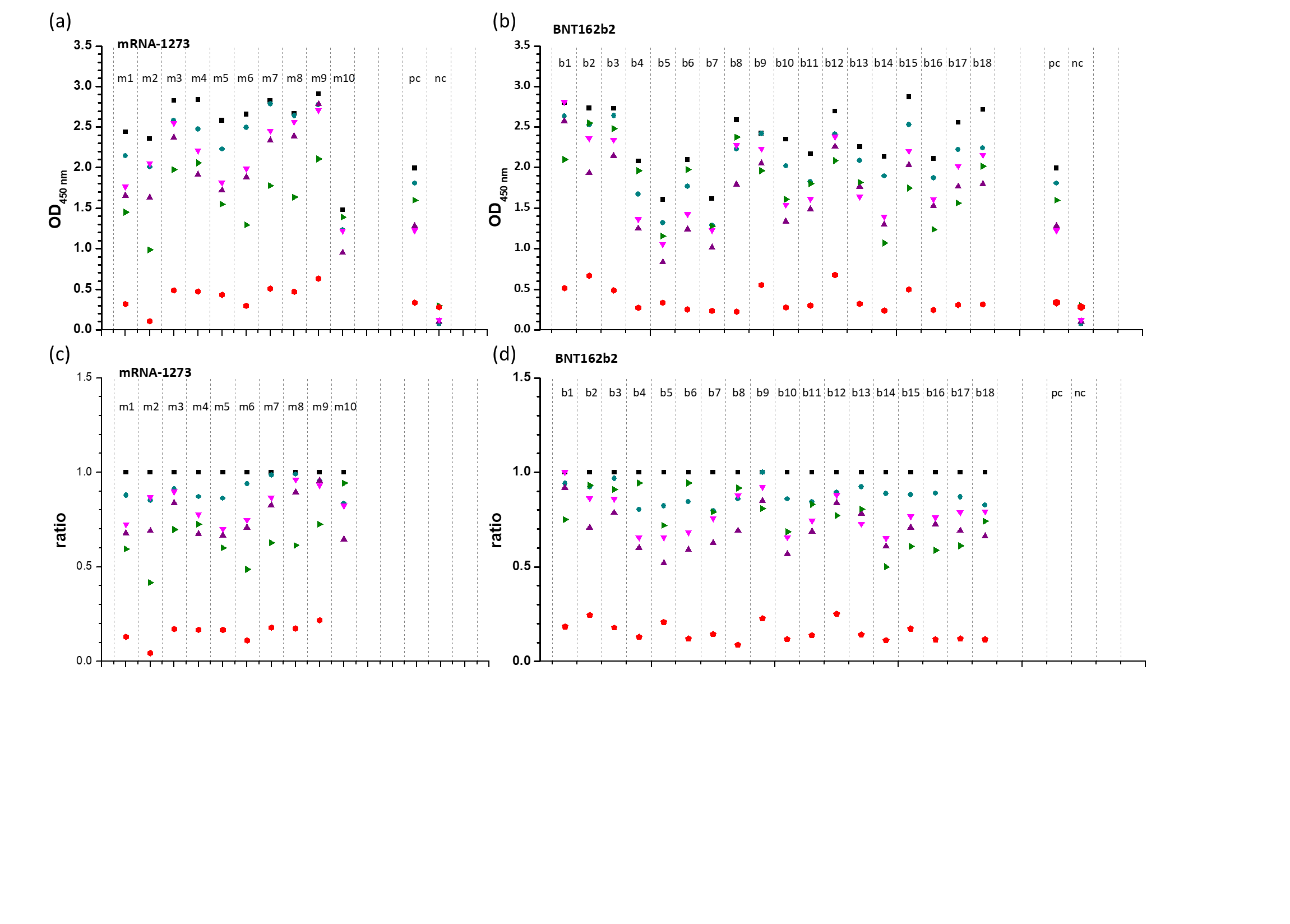


**Figure S7**: Distribution of the OD_450_ values obtained in the anti-RBD IgG ELISA (a, b) for 200-fold diluted serum samples collected from persons after two vaccinations with mRNA-1273 (a, c) or BNT162b2 (b, d) and the same OD_450_ values normalized to the OD_450_ value of the same patient obtained for the wild type RBD (c, d). PC denotes positive sample obtained from a pool of SARS-CoV-2 positive patients and NC denotes a pool of negative control samples collected in the year 2015. Sera were tested against wild type (black square), alpha (blue circle), beta (violet triangle up), gamma (magenta triangle down), delta (green triangle right), and omicron RBDs (red hexagonal).


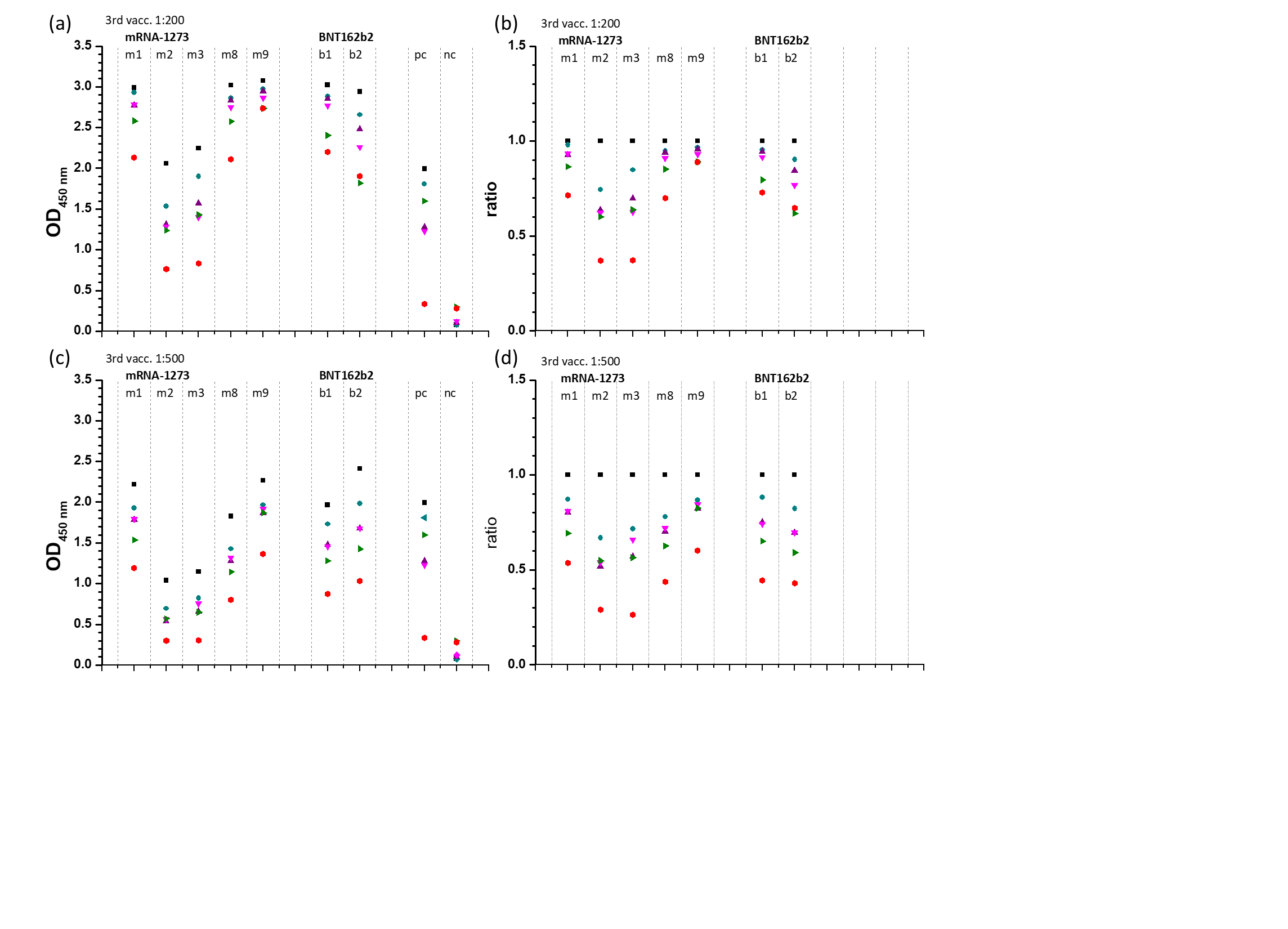


**Figure S8**: Distribution of the OD_450_ values obtained in the anti-RBD IgG ELISA (a, c) for 200-fold (a, b) or 500-fold (c, d) diluted serum samples collected from persons after three vaccinations with mRNA-1273 (a, b) or BNT162b2 (c, d) and the same OD_450_ values normalized to the OD_450_ value of the same patient obtained for wild type RBD (c, d). PC denotes positive sample obtained from a pool of SARS-CoV-2 positive patients and NC denotes a pool of negative control samples collected in the year 2015. Sera were tested against wild type (black square), alpha (blue circle), beta (violet triangle up), gamma (magenta triangle down), delta (green triangle right), and omicron RBDs (red hexagonal).


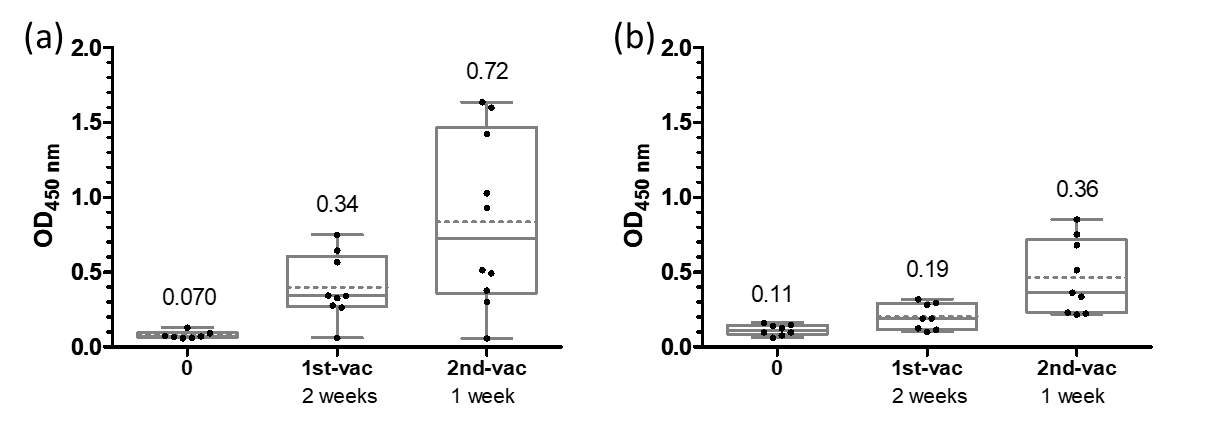


**Figure S9**: Box plots of OD_450_ values obtained in an anti-wt RBD IgA ELISA probed with sera and plasma samples obtained from persons vaccinated with mRNA-1273 (a) or BNT162b2 (b). The median values are provided above the boxes and are additionally indicated as grey horizontal lines in the boxes, while the mean values are shown as grey horizontal dotted lines.

**Figure S10**: OD_450_ values obtained in the inhibition assay for wild type (black square), alpha (blue circle), beta (violet triangle up), gamma (magenta triangle down), delta (green triangle right), and omicron RBDs (red hexagonal) using a dilution series of ACE-2 protein.

**Figure S11**: OD_450_ values obtained in the inhibition assay for wild type RBD using sera from vaccinated patients in two different dilutions (1:100 – blue, 1:10 – black).


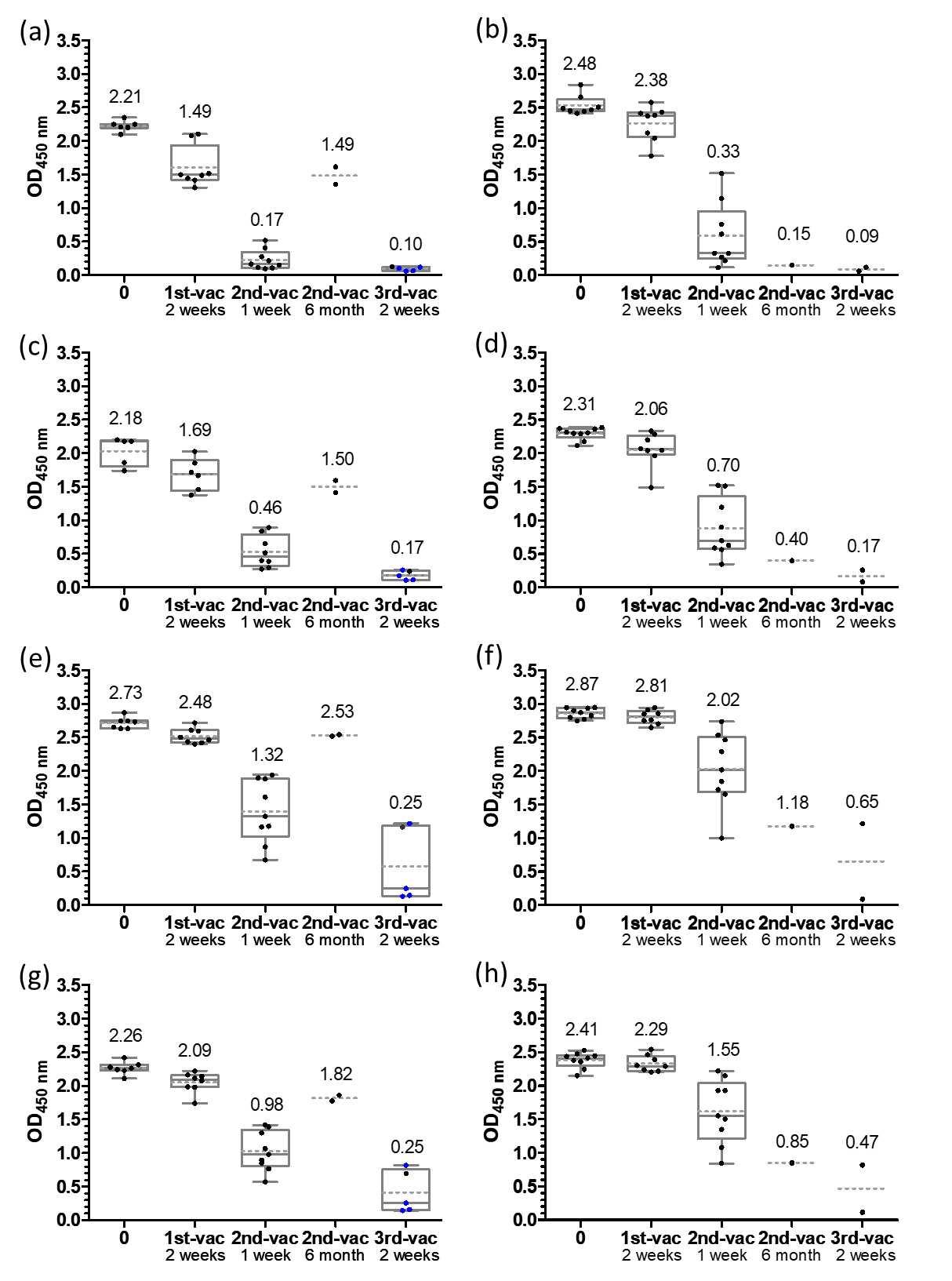


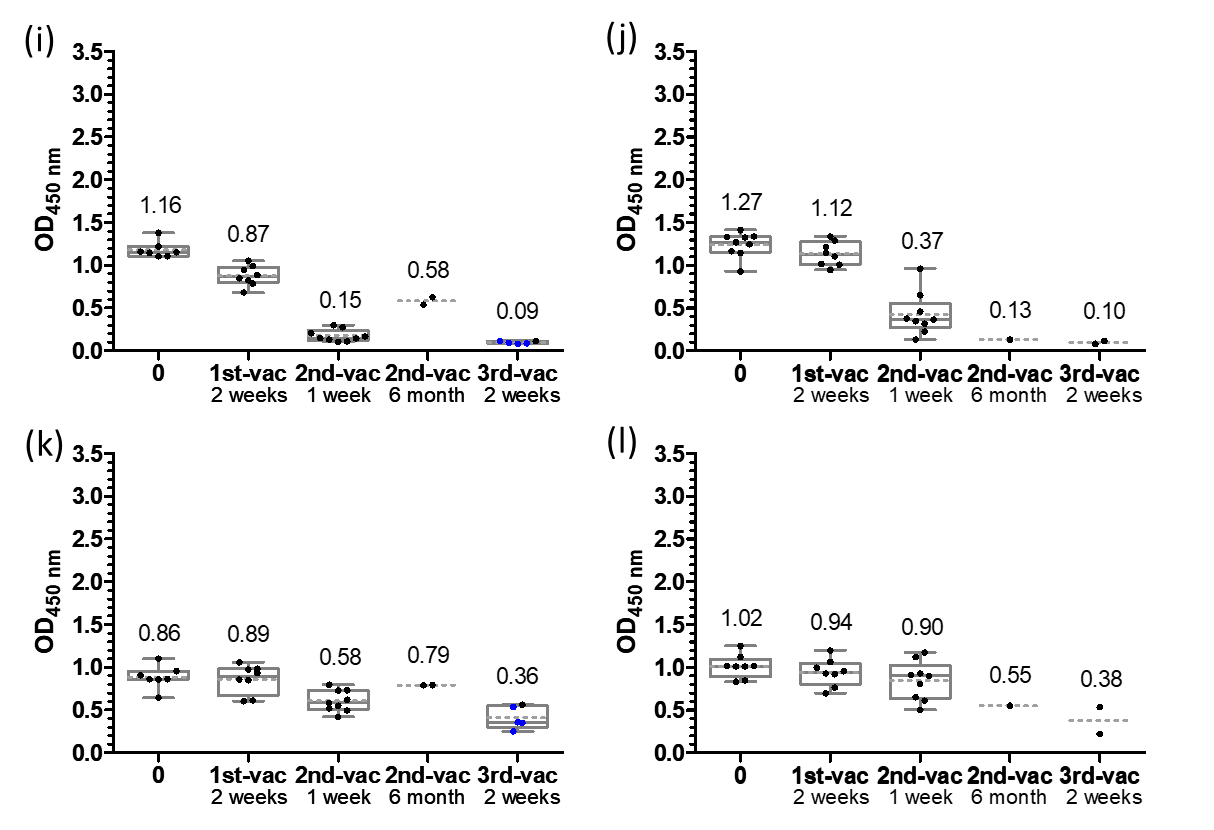


**Figure S12**: Box plots of the OD_450_ values obtained in the inhibition assay using wild type (a, b), alpha (c, d), beta (e, f), gamma (g, h), delta (i, j), and omicron RBDs (k, l). Serum or plasma samples were obtained from persons vaccinated with mRNA-1273 (left panels a to k) or with BNT162b2 (right panels b to l). Sera collected from persons with heterologous booster vaccinations (2x mRNA-1273 and once BNT162b2) are indicated as blue dots. The median values are provided above the boxes and are additionally indicated as grey horizontal lines in the boxes, while the mean values are shown as grey horizontal dotted lines.


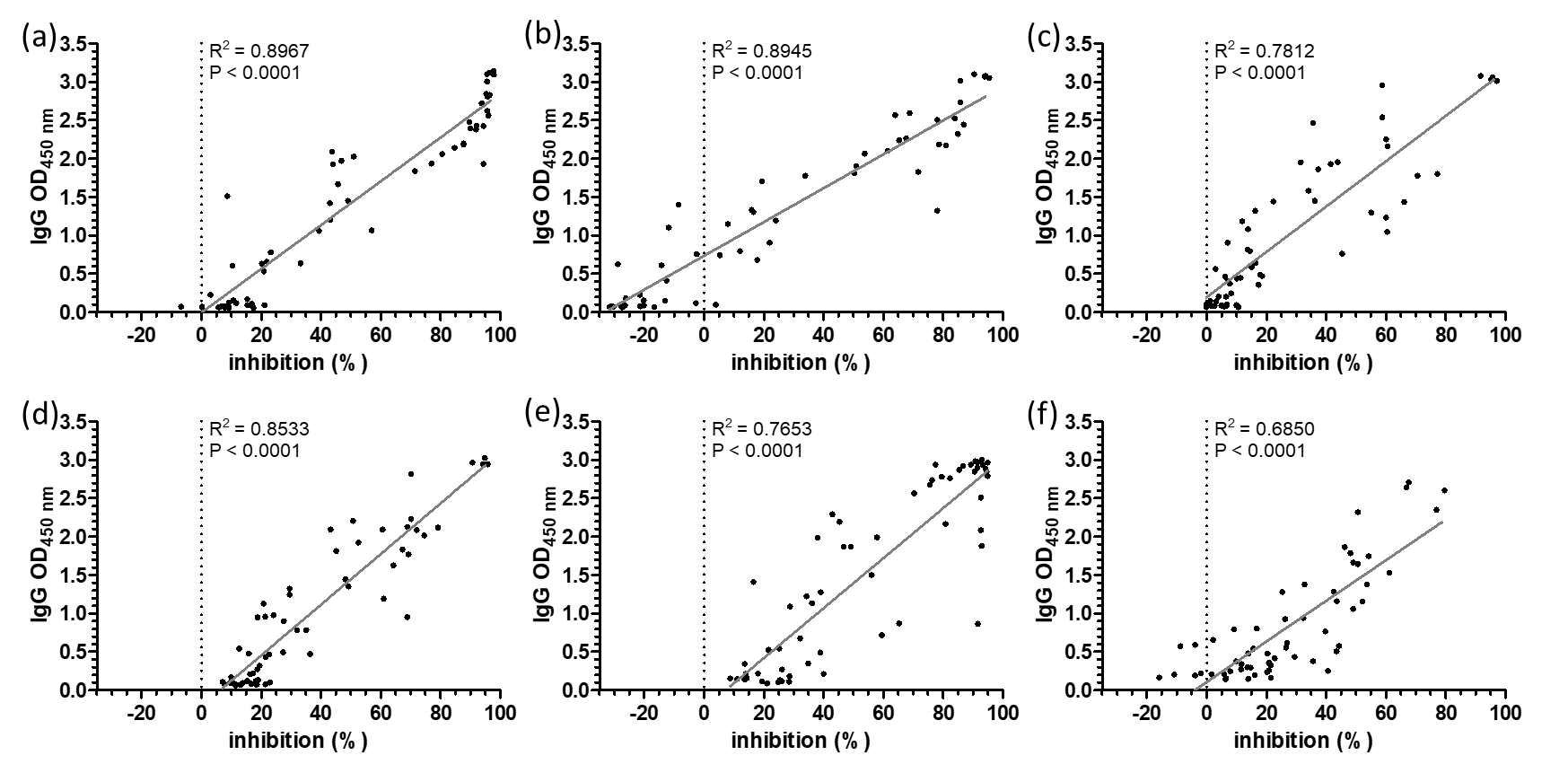


**Figure S13**: Plots indicating the correlation between OD_450_ values obtained in the anti-RBD IgG ELISA and normalized OD_450_ values of the inhibition assay for wild type (a), alpha (b), beta (c), gamma (d), delta (e), and omicron RBDs (f). Linear regression lines are shown in grey, the associated coefficients of determination (R^2^) and p-values are provided on top of each figure.
